# Ultrasound-Stimulated Microbubble Cavitation Combined With Anti-PD-L1 Blockade Inhibits the Progression of MC38 Tumors and Alters the Composition of Gut Microbiota in Mice

**DOI:** 10.1155/ijm/5514372

**Published:** 2025-10-17

**Authors:** Hui Li, Guoliang Yang, Jun Yang, Jiabei Yin, Lei Yao, Yi Zhang, Jingzhen Zhu, Yiyi Liao, Zheng Liu, Ningshan Li

**Affiliations:** ^1^Department of Ultrasound, The Second Affiliated Hospital of Army Medical University, Chongqing, China; ^2^Department of Urology, Urologic Surgery Center, The Second Affiliated Hospital of Army Medical University, Chongqing, China

**Keywords:** gut microbiota, immunotherapies, MC38 tumor, PD-L1 inhibitor, USMC

## Abstract

PD-L1 inhibitor immunotherapies have achieved significant advances in cancer treatment, yet only a subset of patients benefits, with response rates varying widely. Previous studies demonstrated that ultrasonic-stimulated microbubble cavitation (USMC) enhances the antitumor effects of PD-L1 inhibitors, suppressing tumor progression and prolonging survival in murine models. Given the gut microbiome's critical role in antitumor immunity and treatment efficacy, the interplay between USMC, immunotherapy, and gut microbiota remains poorly understood. To investigate this relationship, we conducted 16S rDNA sequencing to analyze the gut microbiota in mice across four treatment groups: control (ck), USMC group (um), PD-L1 inhibitor (pdl1), and USMC+PD-L1 inhibitor (um-pdl1). Our results revealed significant variations in gut microbial composition and abundance among the groups. Notably, we identified a correlation between commensal microbiota profiles and therapeutic responses. Mice treated with pdl1 alone or in combination with um exhibited marked reductions in tumor volume and weight, along with prolonged survival and concurrent shifts in gut microbiota. Multimatrix correlation analysis further identified four bacterial genera (*Akkermansia*, *Bacteroides*, *Escherichia*-*Shigella*, and *Enterococcus*) positively associated with treatment efficacy in the pdl1 and um-pdl1 groups. In summary, our findings preliminarily reveal substantial alterations in gut microbiota following tumor development, with the um-pdl1 regimen exerting a pronounced influence on microbial composition.

## 1. Introduction

Cancer immunotherapy has become a significant therapeutic strategy in oncology, particularly with respect to CTLA-4 and the interaction between programmed cell death protein 1 (PD-1) and its ligand 1 (PD-L1) [1–7], which has been approved for the treatment of several human cancers [5, 6, 8–10]. Although the majority of patients who respond to immune checkpoint blockade (ICB) drugs achieve long-term disease control, approximately one-third of them experience a relapse [8]. Therefore, improving and predicting the efficacy of immunotherapy has become an urgent challenge in the field of tumor immunotherapy.

Studies have demonstrated that ultrasonic-stimulated microbubble cavitation (USMC) plays an important role in regulating the tumor microenvironment and enhancing tumor immunotherapy [11]. USMC affects the tumor immune microenvironment through the induction of microvascular rupture, inducing apoptosis in tumor cells, inhibiting tumor blood vessel formation, and boosting immune responses [12–14]. Additionally, targeted drug and gene delivery can be activated by local ultrasound exposure, resulting in improved therapeutic efficiency [15]. Qiao et al. found that ultrasound can activate tumor metabolic inhibitors, inducing a state of “tumor starvation” that enhances sonodynamic immunotherapy for breast cancer [16]. Our previous research also indicated that the integration of ultrasound with microbubbles may heighten the efficacy of immunotherapy by promoting tumor blood perfusion through USMC, increasing the infiltration of CD8-positive T lymphocytes, and enhancing the administration of PD-L1 inhibitor antibodies. This enhances the PD-L1 blocking effect in mouse MC38 colon cancer, leading to better tumor growth suppression and an extended survival period [17].

A wealth of studies has shown that the gut microbiota is a significant factor influencing the efficacy of tumor immunotherapy [10, 18–23]. Research has demonstrated that the ingestion of certain symbiotic bacteria, when used alongside anti-PD-1/PD-L1 therapies, can nearly arrest the progression of tumors [19, 21]. *Lactobacillus rhamnosus GG (LGG)* can modulate inflammatory responses and inhibit tumor progression during cancer development and transformation [24–26]. *Enterococci* can activate immune signaling pathways and modulate responses related to infection, autoimmunity, and graft-versus-host disease [27–31]. Commensal *Bifidobacterium* contributes to antineoplastic responses and heightens the benefits of PD-L1 inhibitor immunotherapies [19]. Additionally, *Akkermansia muciniphila* stimulates the adaptive immune system in the intestines while maintaining equilibrium [32].

Both USMC and gut microbiota play critical roles in tumor immunotherapy. However, the impact of USMC on gut microbiota, as well as the interplay among these three factors, remains unexplored. To address this knowledge gap, we investigated how USMC and immunotherapy collectively influence gut microbiota composition. Using 16S rRNA gene amplicon sequencing, we analyzed gut microbiota profiles in mice subjected to different tumor intervention strategies. Our findings preliminarily revealed significant variations in microbial community structure and associated metabolic pathways across treatment groups. These microbiota signatures may serve as potential biomarkers for predicting therapeutic response. This study provides novel insights into the tripartite relationship between USMC, immunotherapy, and gut microbiota, offering a foundation for optimizing future tumor immunotherapy strategies.

## 2. Materials and Methods

### 2.1. Cells and Animals

The MC38 murine colon carcinoma cell line (provided by the Oncology Department of Xinqiao Hospital) was cultured in high-glucose Dulbecco's modified Eagle medium (DMEM; Gibco) supplemented with 10% fetal bovine serum (FBS; Gibco) and 100 U/mL penicillin/streptomycin (Beyotime) at 37°C in a humidified 5% CO_2_ atmosphere. A 6–8-week-old male C57BL/6 mice were obtained from the Chinese Academy of Medical Sciences (Beijing, China) and housed under specific pathogen-free conditions maintained at 18°C–26°C with 50%–60% humidity and a 12-h light/dark cycle. Mice were acclimatized under standardized conditions prior to random group assignment at the initiation of experimental treatments. All animals continued to be maintained under identical environmental conditions throughout the study period. All animal procedures were performed in accordance with the Animal Study Guidelines of Army Medical University and were approved by the Institutional Animal Care and Use Committee (IACUC) of Army Medical University (Protocol No. AMUWEC20224334). The study protocol received additional review and approval by the Laboratory Animal Welfare and Ethics Committee of Third Military Medical University.

### 2.2. Murine Oncology Model

An MC38 cell suspension was prepared in phosphate-buffered saline (PBS) at a concentration of 5 × 10^6^ cells/mL. A 100 *μ*L aliquot of this suspension was injected subcutaneously into the right flank of male C57BL/6 mice.

### 2.3. Treatment Protocols

The USMC therapy was delivered using a VINNO70 diagnostic ultrasound system equipped with a specialized Vflash mode designed to modulate microbubble cavitation parameters, including frequency, mechanical index (MI), pulse length (PL), pulse repetition frequency (PRF), and pulse/interval duration.

For cavitation induction, 0.04 mL of Zhifuxian (a proprietary microbubble formulation) [33] was diluted in 0.4 mL saline and slowly infused through the tail vein during USMC treatment. Ultrasound parameters were set as follows: X4-12 transducer operating at 3 MHz frequency, MI of 0.3, PL of 18 cycles, and PRF of 2 kHz. Using phased focusing, the therapeutic ultrasound beam was targeted to a specific region of interest (ROI) with an intermittent duty cycle of 1-s exposure followed by 1-s rest, repeated continuously for 10 min.

Twenty-four MC38 tumor-bearing mice with tumor volumes of 150–200 mm^3^ were randomly divided into four experimental groups (*n* = 6 per group): ck (blank control), um (USMC treatment alone), pdl1 (anti-PD-L1 antibody treatment alone), and um-pdl1 (combined USMC and anti-PD-L1 antibody treatment). Mice received USMC treatment in the tumor region every 3 days for a total of four sessions. PD-L1 inhibitor antibody (Bio-X-cell, BE0101, 200 *μ*g) was administered intraperitoneally 24 h after each USMC treatment. Fecal samples were collected before treatment initiation, during the treatment period, and after treatment completion. For each collection timepoint, two to three fecal pellets were collected per mouse, placed in sterile centrifuge tubes, flash-frozen in liquid nitrogen, and stored at −80°C. Tumor volume was calculated using the following formula: (long axis) × (short axis)^2^ × *π*/6. Body weight and tumor weight were measured posttreatment. Survival was monitored for 70 days. Following anesthesia with 1% pentobarbital sodium, all mice were humanely euthanized via CO_2_ asphyxiation.

### 2.4. 16S rRNA Sequencing and Analysis

Fecal DNA was extracted from mice across the ck, um, pdl1, and um-pdl1 groups at various time points utilizing a fecal DNA extraction kit (DP712, Tiangen Company, Beijing, China). The procedure was conducted with three biological replicates for each group. The V3–V4 hypervariable region of the 16S rDNA gene was targeted for amplification via PCR with the primers 341F (CCTACGGGNGGCWGCAG) and 806R (GGACTACHVGGGTATCTAAT/GACTACHVGGGTATCTAATCC), incorporating a unique eight-base barcode for each sample [34]. We prepared sequencing libraries following the Illumina TruSeq DNA PCR-Free protocol and incorporated unique index codes. Sequencing was executed on the Illumina NovaSeq 6000 system provided by Genecloud Co. Ltd., Chongqing, China [34]. Analysis of the sequences was performed with the QIIME software package, Version 2.0, Release 2020.2 [34]. Operational taxonomic units (OTUs) were identified from effective tags with a similarity threshold of 97% or higher, utilizing the MOTHUR pipeline. The alpha diversity of bacteria was ascertained through a sampling-based OTU analysis, with results depicted as observed OTUs, the Chao estimator, Shannon diversity index, and Simpson diversity index, all computed with the “vegan” package in the R programming environment [34]. Microbiome beta diversity among samples was evaluated through principal coordinate analysis (pCoA) utilizing the R statistical package. The analysis incorporated various metrics, including Bray–Curtis, unweighted and weighted UniFrac distances, calculated through the phyloseq package. Comparative taxonomic assessments of bacterial groups—spanning phyla, classes, orders, families, and genera—were performed between pairs of groups, employing the Wilcoxon rank-sum test. Furthermore, the differential abundance of bacterial communities was examined with the LEfSe method, leveraging linear discriminant analysis (LDA) effect size for statistical inference (http://huttenhower.sph.harvard.edu/lefse/) [34]. The relationship between gut microbiota and metabolites was analyzed using Pearson's correlation analysis, employing the mixOmics package in R to calculate the coefficient of determination (*r*^2^) and the corresponding *p* values. Multiple detection corrections were applied during the analysis of the microbial community. The 16S rRNA gene sequences referenced in this publication are archived in the Genome Sequence Archive within the journal *Genomics, Proteomics & Bioinformatics* for the year 2021 and are also deposited at the National Genomics Data Center, as cited in Nucleic Acids Research for 2022. These sequences, with the Accession Number CRA011626 (https://bigd.big.ac.cn/gsa/browse/CRA011626), can be accessed through the China National Center for Bioinformation/Beijing Institute of Genomics, Chinese Academy of Sciences, and are available online.

### 2.5. Cooccurrence Network Analysis

We elucidated the relationships among bacterial genera by creating a cooccurrence network from 16S rRNA data. The network was developed using Spearman's correlation coefficient to assess the linkages between genera present in samples categorized as ck, um, pdl1, and um-pdl1, based on their relative abundances. Notable correlations were identified with a significance threshold of *p* < 0.05 and a correlation strength of *r* ≥ 0.7, and these were graphically represented using Gephi 0.9.7. The structural similarity of the networks was evaluated through node closeness and the overlap of correlations. Node centrality, indicative of a node's importance within the network, was determined using Gephi. Shared correlations were identified as edges connecting identical nodes across both networks. For inclusion in the analysis, a genus had to be detected in a minimum of 10% of the samples.

Partial Mantel tests were utilized to evaluate correlations between variations in gut microbiota and metabolic pathways, as well as tumor metrics including volume and weight, alongside survival and body weight data.

### 2.6. Statistical Analysis

Results are presented as the average with the standard error of the mean (SEM) indicated. Graphs were crafted with GraphPad Prism software, Version 8.0, and statistical evaluations were performed using SPSS, Version 26.0. A threshold of *p* < 0.05 was set to denote statistical significance. Normality of the data distribution was confirmed with the Shapiro–Wilk test. For pre- and posttreatment comparisons, paired *t*-tests were applied, while one-way ANOVA served for assessing differences across multiple groups, followed by post hoc analysis using the least significant difference method for pairwise comparisons. For nonnormally distributed data, the Mann–Whitney *U* test was implemented, with statistical significance also defined as *p* < 0.05.

## 3. Results

### 3.1. PD-L1 Inhibitor and USMC+PD-L1 Inhibitor Significantly Inhibited MC38 Tumor Growth in Mice

**Figure 1:**
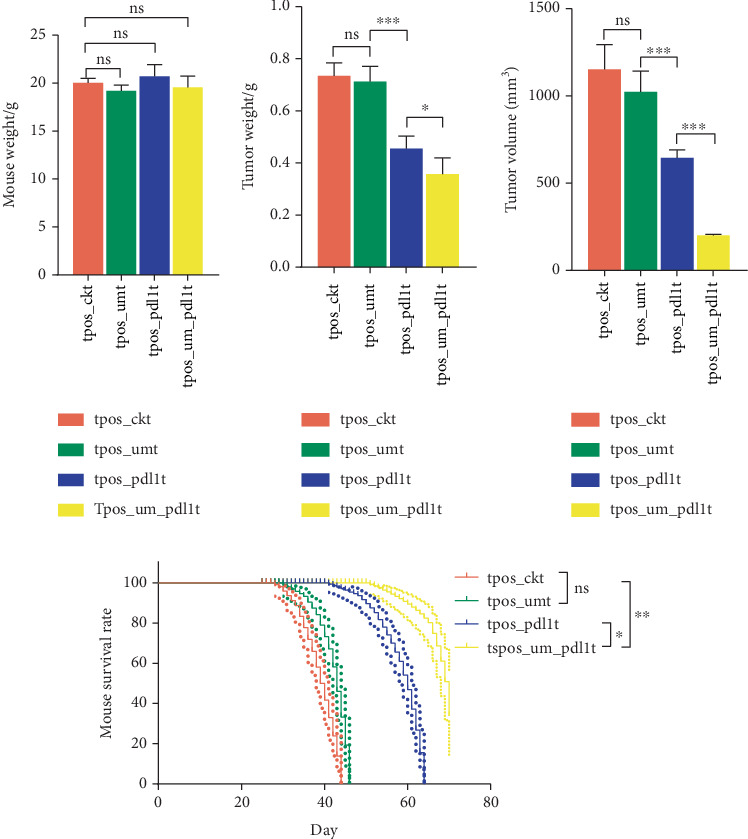
Comparison of mouse weight, tumor weight, tumor volume, and mouse survival rate between different treatment groups. (a) Bar graph of mouse weight. (b) Bar graph of tumor weight. (c) Bar graph of tumor volume. (d) Plot of mouse survival. ⁣^∗^*p* < 0.05, ⁣^∗∗^*p* < 0.01, and ⁣^∗∗∗^*p* < 0.001.

In this study, we investigated three therapeutic approaches for MC38 tumors in mice: USMC alone, PD-L1 inhibitor monotherapy, and their combination. We systematically assessed changes in body weight, tumor volume, tumor mass, and survival duration across all experimental groups (Figure 1 and Supporting Information 2: Table S1). No significant differences in body weight were observed among groups (Figure 1a). Tumor volume, mass, and survival time in the USMC-treated group (tpos-umt) showed no notable improvement compared to controls (tpos-ckt) (Figures 1b, 1c, and 1d). Both PD-L1 inhibitor monotherapy (tpos-pdl1t) and combination therapy (tpos-um-pdl1t) groups exhibited significant reductions in tumor volume (*p* < 0.001) and mass (*p* < 0.001) (Figure 1b,c) and prolonged survival (*p* < 0.01) compared to ck and USMC-only groups (Figure 1d). Critically, the combination therapy demonstrated superior efficacy to PD-L1 inhibitor alone, with further reductions in tumor volume (*p* < 0.001) and mass (*p* = 0.012), as well as extended survival (*p* = 0.041). USMC alone neither inhibits tumor progression nor enhances survival in this model. In contrast, PD-L1 blockade—both as monotherapy and combined with USMC—significantly attenuates tumor growth and improves survival. The synergistic effect of USMC with PD-L1 inhibition suggests potential clinical relevance for combination strategies.

### 3.2. MC38 Tumor Could Disrupt the Structure of Gut Microbiota and KEGG Pathways in Mice

**Figure 2:**
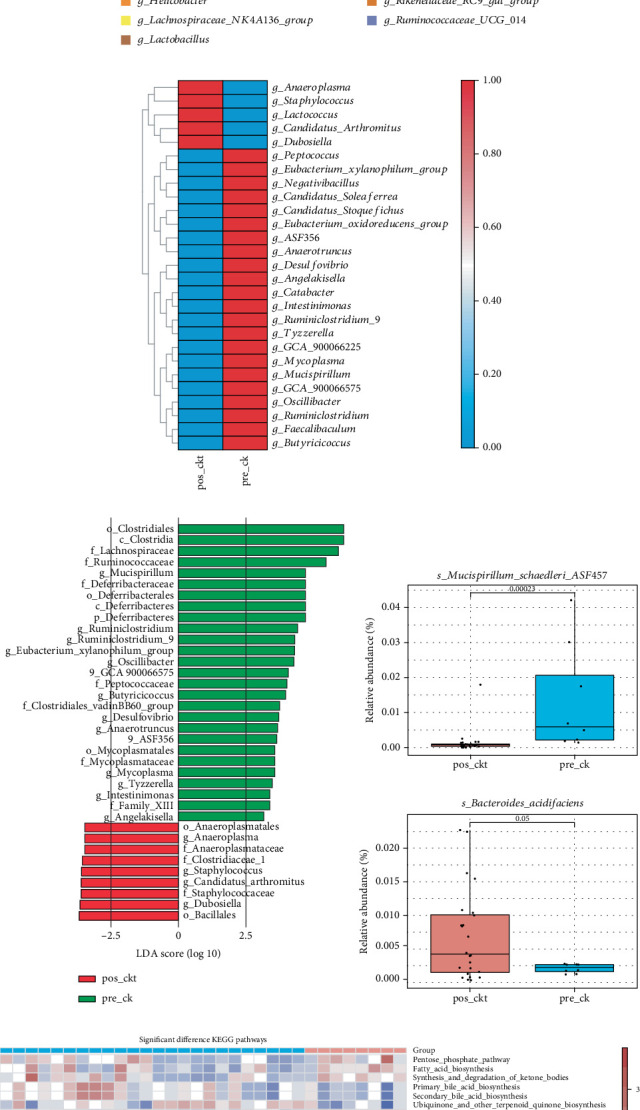
Changes of intestinal flora in mice before and after implantation tumor. (a) ANOSIM-analysis results represent the difference between groups; others are within groups; the greater the distance is, the greater the difference is; and the thickness is the sample size. (b) The clustering analyses of partial least-squares discriminant analysis (PLS-DA). (c) Alpha diversity (based on the ACE, Chao1, and Shannon indices). (d, e) Relative abundance of the indicated phylum and genus in pos-ckt and pre-ck. (f) Heatmap of selected differentially abundant genus in pos-ckt and pre-ck. (g) LDA scores for the bacterial taxa showed different abundant between pos-ckt and pre-ck (LDA score > 2.5). (h) The Top 20 of differentially abundant analysis between pos-ckt and pre-ck at the level of species (*p* < 0.05). (i) KEGG pathway of differentially altered metabolites between before and after of treatment. (j) Correlation heatmap of differentially abundant gut microbiota and differentially altered metabolites (*p* < 0.05) from the pos-ckt and pre-ck. Blue represents a positive correlation, red represents a negative correlation, and a darker color corresponds to a stronger correlation. ⁣^∗^*p* < 0.05, ⁣^∗∗^*p* < 0.01, and ⁣^∗∗∗^*p* < 0.001.

To comprehensively assess the impact of MC38 tumors on murine gut microbiota, we conducted comparative analyses of intestinal microbial communities at two critical time points: prior to and following tumor establishment. Our analysis revealed significant *β*-diversity differences between pretumor (pre-ck, *n* = 8) and posttumor (pos-ckt) groups (ANOSIM: *R* = 0.472, *p* = 0.001), indicating tumor-induced microbial community restructuring (Figure 2a). Distinct clustering of microbial profiles was confirmed by PLS-DA (Figure 2b). Marked reduction in *α*-diversity in tumor-bearing mice, as evidenced by significantly decreased ACE, Chao1, and Shannon indices (*p* < 0.01, Figure 2c). At the phylum level, *Firmicutes*, *Bacteroidetes*, *Epsilonbacteraeota*, *Verrucomicrobia*, and *Deferribacteres* were predominant across the different groups (Figure 2d). At the genus level, *g_Lachnospiraceae_NK4A136_group*, *g_Lactobacillus*, *g_Prevotellaceae_UCG_001*, *g_Rikenellaceae_RC9_gut_group*, *g_Akkermansia*, *g_Prevotellaceae_NK3B31_group*, and *g_Alistipe*s were dominant among the various groups (Figure 2e). Specifically, the pos-ckt group showed a significant decrease in the abundance of *g_Peptococcus*, *g_Eubacterium_xylanophilum_group*, *g_Negativibacillus*, *g_Candidatus_Soleaferrea*, *g_Candidatus_Stoquefichu*s, *g_Eubacterium_oxidoreducens_group*, *g_ASF356*, *g_Anaerotruncus*, *g_Desulfovibrio*, *g_Angelakisella*, *g_Catabacter*, *g_Intestinimonas*, *g_Ruminiclostridium_9*, *g_Tyzzerella*, *g_GCA_900066225*, *g_Mycoplasma*, *g_Mucispirillum*, *g_GCA_900066575*, *g_Oscillibacter*, *g_Ruminiclostridium*, *g_Faecalibaculum*, and *g_Butyricicoccus*, while *g_Anaeroplasma*, *g_Staphylococcus*, *g_Lactococcus*, *g_Candidatus_Arthromitus*, and *g_Dubosiella* were significantly increased compared to the pre-ck group (Figure 2f). At the species level, *s_Mucispirillum_schaedleri_ASF457* significantly decreased, whereas *s_Bacteroides_acidifaciens* significantly increased in the pos-ckt group (Figure 2h). The LDA plot (LDA score > 2.5), distinctly revealed microbiota composition changes in the pos-ckt group (Figure 2g) and clearly illustrated alterations in the microbiota of the pos-ckt group (Figure 2g). In terms of specific changes, at the family level, the pos-ckt group showed enrichment in families such as *f_Anaeroplasmataceae*, *f_Clostridiaceae_1*, and *f_Staphylococcaceae*, while the pre-ck group contained more *f_Lachnospiraceae*, *f_Ruminococcaceae*, *f_Deferribacteraceae*, *f_Peptococcaceae*, *f_Clostridiales_vadinBB60_group*, *f_Mycoplasmataceae*, and *f_Family_XIII*. At the taxonomic level of genus, *g_Anaeroplasma*, *g_Staphylococcus*, *g_Candidatus_Arthromitus*, and *g_Dubosiella* were more abundant in the pos-ckt group, whereas *g_Mucispirillum*, *g_Ruminiclostridium*, *g_Ruminiclostridium_9*, *g_Eubacterium_xylanophilum_group*, *g_Oscillibacter*, *g_GCA_900066575*, *g_Butyricicoccus*, *g_Desulfovibrio*, *g_Anaerotruncus*, *g_ASF356*, *g_Mycoplasma*, *g_Tyzzerella*, *g_Intestinimonas*, and *g_Angelakisella* were more abundant in the pre-ck group. In summary, the microbial composition of the pos-ckt group showed alterations compared to the pre-ck group, further indicating that gut microbiota dysbiosis occurs when MC38 tumors are inoculated into mice.

All significantly differentiated KEGG pathways were systematically evaluated (Figure 2i). Spearman's rank correlation analysis revealed key associations between gut microbiota shifts and metabolic pathway alterations (Figure 2j). The significant enrichment of gut microbiota in the pos-ckt group, such as *g_Anaeroplasma*, *g_Staphylococcus*, *g_Candidatus_Arthromitus*, *g_Lactococcus*, and *g_Dubosiella*, was demonstrated positive associations with several metabolic pathways, including the production of primary and secondary bile acids, the biosynthesis involved in Chagas disease, the synthesis of ubiquinone and other terpenoid quinones, and the formation of lipopolysaccharides. In contrast, the gut microbiota enrichment in the pre-ck group showed significant positive correlations with a range of metabolic processes. These included the pentose phosphate pathway, fatty acid synthesis, ketone body synthesis and breakdown, cysteine and methionine metabolism, and the biosynthesis of valine, leucine, and isoleucine. Additionally, it correlated with D-arginine and D-ornithine metabolism, lysine production, porphyrin and chlorophyll metabolism, pantothenate and CoA biosynthesis, and the formation of unsaturated fatty acids. The microbiota was also linked to ansamycin biosynthesis, the function of ABC transporters, two-component systems, bacterial chemotaxis, flagellar assembly, and mRNA surveillance mechanisms. Other correlated pathways comprised RNA polymerase activity, nonhomologous end joining, insulin signaling, chlorocyclohexane and chlorobenzene degradation, and phenylalanine metabolism. In conclusion, the gut microbiota and its metabolic pathways were significantly altered in mice before and after MC38 tumor implantation, indicating that the occurrence and progression of tumors can profoundly affect the gut microbiota in mice.

### 3.3. Effects of Different Tumor Intervention Schemes on Gut Microbiota in Mice

**Figure 3:**
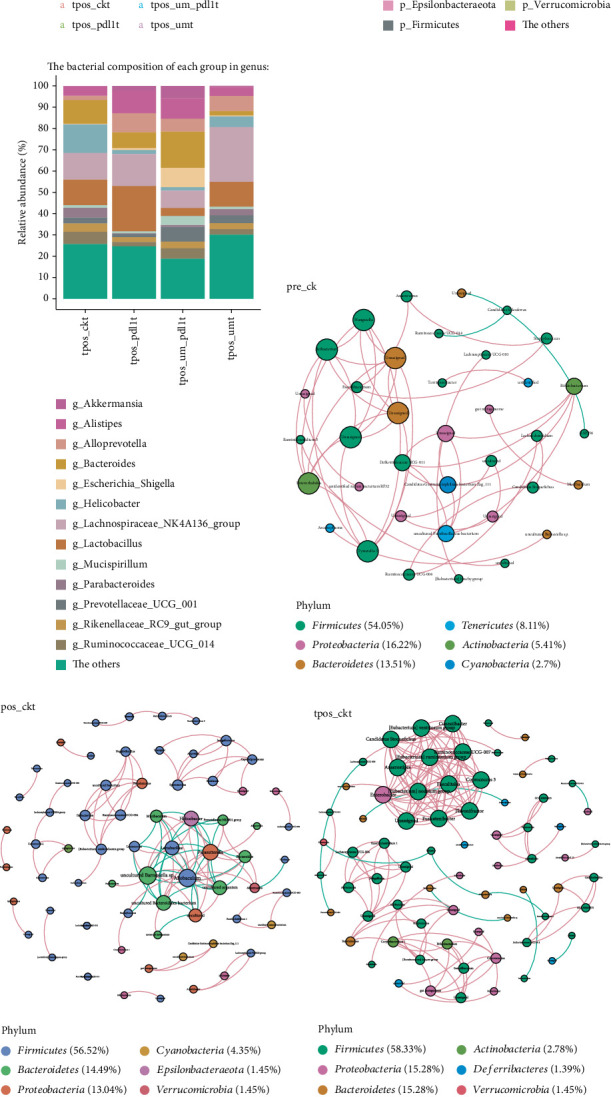
Diversity of gut microbiota are different group. (a–c) Alpha diversity (based on the ACE, Shannon, and Chao1 indices). (d) PCoA of the beta diversity metric distance. (e) The unweighted pair group method with arithmetic mean algorithm was used to construct the sample clustering tree, with different colors representing different groups and branch lengths representing the distance between samples. (f, g) The mean percentages of each community contributed by the indicated phylum (higher than 0.1% are shown) and genus (higher than 1% are shown). (h–m) Genera cooccurrence network between pre-ck, pos-ckt, tpos-ckt, tpos-umt, tpos-pdl1t, and tpos-um-pdl1t group based on the Spearman correlation algorithms. Each node presents a bacterial genus. The node size indicates the weighted value of each genus per group, and the thickness of the line represents the Spearman coefficient (*p* < 0.05, *r* ≥ 0.7). ⁣^∗^*p* < 0.05, ⁣^∗∗^*p* < 0.01, and ⁣^∗∗∗^*p* < 0.001.

16S rRNA sequencing analysis revealed significant gut microbiota alterations induced by different tumor treatment strategies in mice. Alpha diversity assessment (Shannon, Chao1, and ACE indices) demonstrated comparable trends across all groups. While the treatment groups (tpos-umt, tpos-pdl1t, and tpos-um-pdl1t) showed modest increases in diversity indices compared to controls (tpos-ckt), these differences were not statistically significant (Figures 3a, 3b, and 3c). A PCoA was applied utilizing the Bray–Curtis dissimilarity index, which distinctly revealed that the microbial profiles of the tpos-ckt and tpos-umt cohorts were closely aligned. In contrast, the tpos-pdl1t and tpos-um-pdl1t cohorts exhibited markedly divergent microbial compositions. Significantly, the analysis indicated that the tpos-pdl1t group's microbial profile closely mirrored that of the tpos-um-pdl1t group (Figure 3d). Sample cluster evolutionary tree analysis corroborated these findings, showing the same results (Figure 3e).

Next, we conducted pairwise ANOSIM analysis for the tpos-ckt, tpos-umt, tpos-pdl1t, and tpos-um-pdl1t groups. The findings distinctly showed no substantial variation between the microbial compositions of the tpos-ckt and tpos-umt groups, just as there was no notable divergence observed between the tpos-pdl1t and tpos-um-pdl1t groups. Nevertheless, pronounced disparities were detected in the microbial profiles when comparing the tpos-ckt with the tpos-pdl1t groups, the tpos-umt with the tpos-pdl1t groups, and additionally between the tpos-um-pdl1t and the tpos-pdl1t groups. Additionally, PD-L1 inhibitor immunotherapy had a significant impact on the gut microbiota, underscoring the significance of our study's groupings (Supporting Information 1: Figures S1a, S1b, S1c, S1d, S1e, and S1f). Upon examining the makeup and relative abundance of microbial groups at the phylum level (relative abundance exceeding 0.1%) and genus level (relative abundance exceeding 1%), we identified notable variations in the gut microbiota composition and relative abundance among mice subjected to various oncological intervention strategies (Figure 3f,g). Significantly, the levels of *g_Akkermansia* and *g_Escherichia_Shigella* were notably elevated in the tpos-pdl1t group, whereas *g_Akkermansia* and *g_Bacteroide*s showed substantial increases in the tpos-um-pdl1t group (Supporting Information 1: Figures S1g, S1h, S1i, S1j, S1k, and S1l). Previous studies have reported that *g_Akkermansia* contributes to the response to PD-L1 inhibitor therapy [32]. Additionally, the LDA effect size (LDA scores exceeding 2.5) revealed a pronounced shift in the microbial community between the tpos-pdl1t and tpos-um-pdl1t groups when juxtaposed with the tpos-ckt and tpos-umt groups (Supporting Information 1: Figures S1m, S1n, S1o, S1p, S1q, and S1r). Moreover, this finding suggests that the USMC did not exert a substantial impact on the intestinal microbiome of the mice, in contrast to the PD-L1 inhibitor immunotherapy, which had a significant effect on the gut microbiota. Whether these changes in gut microbiota enhance the efficacy of immunotherapy requires further in-depth investigation. Overall, the results showed significant changes in the gut microbiota of mice subjected to different tumor intervention schemes.

A prior study indicates that the diverse microbial environment of the human intestine is selectively influenced by different species of microbes within its communities [35]. To investigate possible associations among gut bacteria, we developed cooccurrence networks for the genera in each group, utilizing significant Spearman correlation coefficients. The pre-ck group predominantly displayed a cooccurrence network characterized by a variety of genera that were derived from six principal phyla, namely, Firmicutes, Proteobacteria, Bacteroidetes, Tenericutes, Actinobacteria, and Cyanobacteria. This network displayed strong positive correlations among genera (Figure 3h). In contrast, as shown in Figures 3i, 3j, 3k, 3l, and 3m, the microbiome of mice after tumor implantation exhibited a more complex network. Compared to the pre-ck group, the other groups demonstrated significantly increased correlations within the microbiome, particularly negative correlations. In order to measure these differences, we calculated the average degree of edges (connections) in the six microbial networks: pre-ck (2.60), pos-ckt (3.28), tpos-ckt (4.17), tpos-umt (2.68), tpos-pdl1t (3.83), and tpos-um-pdl1t (3.07). Overall, these studies indicate that the intestinal microbiome makeup and prevalence in mice are shaped by various oncological intervention approaches.

### 3.4. Changes of Gut Microbiota Before and After Different Tumor Intervention Schemes

**Figure 4:**
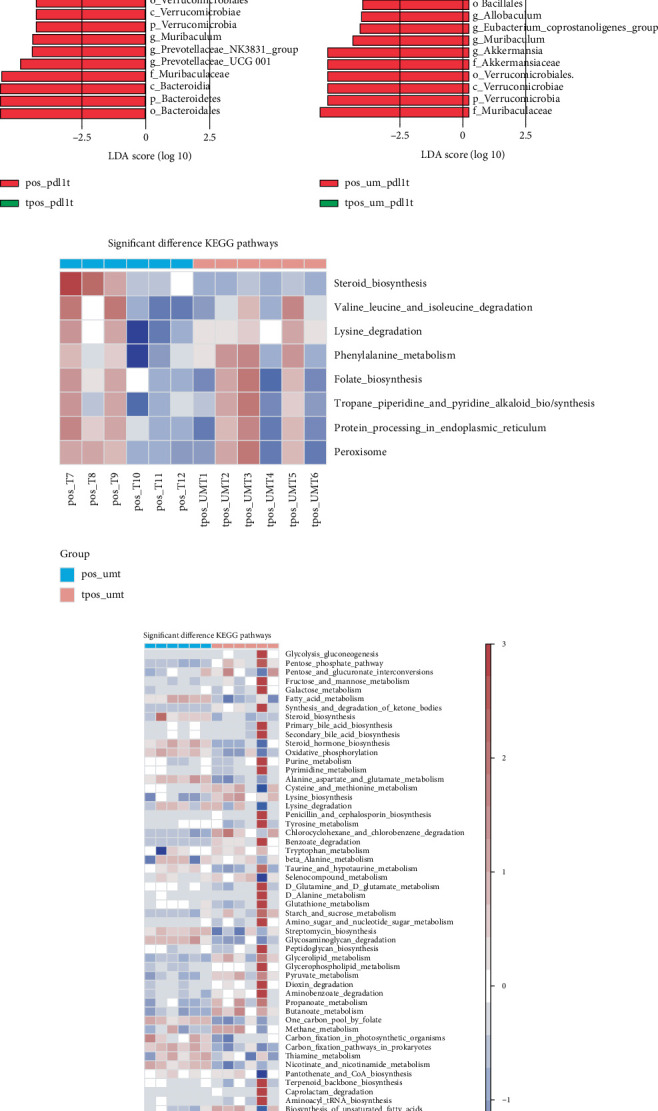
Changes of intestinal flora in mice before and after treatment with different tumor intervention schemes. (a–c) Alpha diversity based on the Simpson index. (d–f) PCoA of the beta diversity metric distance. (g–i) The mean percentages of each community contributed by the indicated genus (higher than 1% are shown). (j–l) LDA scores for the bacterial taxa showed different abundant between before and after of treatment (LDA score > 2.5). (m–o) KEGG pathway of differentially altered metabolites between before and after of treatment. (p–r) Correlation heatmap of differentially abundant gut microbiota and differentially altered metabolites (*p* < 0.05) from the before and after of treatment. Blue represents a positive correlation, red represents a negative correlation, and a darker color corresponds to a stronger correlation. ⁣^∗^*p* < 0.05, ⁣^∗∗^*p* < 0.01, and ⁣^∗∗∗^*p* < 0.001.

To characterize treatment-induced gut microbiome changes in tumor-bearing mice, we collected longitudinal fecal samples from four experimental groups (ck, um, pdl1, and um-pdl1) for 16S rRNA gene sequencing analysis. We assessed alpha diversity using the Simpson index (Figures 4a, 4b, and 4c). As indicated in these figures, the um, pdl1, and um-pdl1 groups exhibited similar trends, with all groups showing an increase in microbiota *α* diversity after treatment compared to before treatment; however, these differences were not statistically significant. A PCoA was performed to assess the resemblance of microbial populations between the two groups, utilizing the Bray–Curtis dissimilarity index as a measure (Figures 4d, 4e, and 4f). This analysis clearly demonstrated differences in microbiota composition before and after treatment with various intervention schemes, particularly with PD-L1 inhibitor immunotherapy alone and USMC combined with PD-L1 inhibitor immunotherapy. By conducting a comparative examination of the gut microbiome's composition and relative abundance (focusing on taxa with relative abundance exceeding 1%), both pretreatment and posttreatment periods were evaluated; we observed significant changes in the gut microbiota following PD-L1 inhibitor immunotherapy (pos-pdl1t [after tumor implantation and before PD-L1 inhibitor treatment] vs. tpos-pdl1t) and USMC combined with PD-L1 inhibitor immunotherapy (pos-um-pdl1t [after tumor implantation and before USMC+PD-L1 inhibitor treatment] vs. tpos-um-pdl1t). In contrast, changes in the USMC alone group (pos-umt [after tumor implantation and before USMC treatment] vs. tpos-umt) were minimal (Figures 4g, 4h, and 4i). Furthermore, the LDA effect size measurements (where LDA scores surpassed 2.5) exposed distinct shifts in the microbial community pre- and postintervention (Figures 4j, 4k, and 4l). Specific genera, such as *g_Lactobacillus*, *g_Blautia*, *g_Oscillibacter*, *g_Mucispirillum*, *g_Escherichia_Shigella*, *g_Ruminiclostridium_5*, *g_Turicibacter*, *g_Candidatus_Saccharimonas*, *g_Negativibacillus*, *g_Candidatus_Soleaferrea*, *g_Ruminococcus_torques_group*, *g_Bilophila*, and *g_Enterococcus*, could serve as biomarkers for the tpos-pdl1t group. In the tpos-um-pdl1t group, potential biomarkers included *g_Escherichia_Shigella*, *g_Romboutsia*, *g_Turicibacter*, *g_Clostridium_sensu_stricto_1*, *g_Enterorhabdus*, and *g_Enterococcus*. For the tpos-umt group, biomarkers included *g_Eubacterium_xylanophilum_group*, *g_Mucispirillum*, *g_Tyzzerella*, *g_Lachnoclostridium*, *g_Butyricicoccus*, *g_Allobaculum*, *g_Intestinimonas*, *g_Erysipelatoclostridium*, *g_Romboutsia*, *g_Eubacterium_oxidoreducens_group*, *g_UBA1819*, and *g_Ruminococcaceae_UGG_009*. Notably, our findings indicated that the relative abundance of *g_Akkermansia* was more pronounced prior to treatment, with a subsequent decline posttreatment. However, its abundance remained markedly higher relative to the tpos-ckt and tpos-umt groups, hinting that a certain level of its relative abundance could be advantageous for oncological therapies. We additionally conducted a KEGG pathway analysis to evaluate the relative abundance of metabolic pathways linked to the gut microbiome. As shown in Figures 4m, 4n, and 4o, the dominant enriched metabolic pathways in the tpos-pdl1t and tpos-um-pdl1t groups were highly consistent, while the dominant metabolic pathways in the tpos-umt group differed significantly from those in the tpos-pdl1t and tpos-um-pdl1t groups. Following this, we conducted correlation matrix analysis for the significantly different gut microbiota and KEGG pathways, generating a correlation matrix using the Spearman correlation (Figures 4p, 4q, and 4r). The dominant metabolic pathways in the tpos-pdl1t and tpos-um-pdl1t groups exhibited significant positive correlations with *g_Escherichia_Shigella*, *g_Aquabacterium*, *g_Lactobacillus*, *g_Candidatus_Saccharimonas*, *g_Turicibacter*, *g_Ruminiclostridium_5*, *g_Oscillibacter*, *g_Negativibacillus*, *g_Candidatus_Soleaferrea*, *g_Ruminococcus_torques_group*, *g_Blautia*, *g_Enterococcu*s, *g_Mucispirillum*, *g_Romboutsia*, *g_Clostridium_sensu_stricto_1*, and *g_Staphylococcus*. In contrast, the dominant metabolic pathways in the tpos-umt group were significantly positively correlated with *g_Romboutsia*, *g_Allobaculum*, *g_Erysipelatoclostridium*, *g_UBA1819*, *g_Ruminococcaceae_UCG_009*, *g_Intestinimonas*, *g_Eubacterium_xylanophilum_group*, *g_Eubacterium_oxidoreducens_group*, *g_Tyzzerella*, *g_Lachnoclostridium*, and *g_Mucispirillum*. Collectively, these analyses suggest that the microbial and metabolic pathway relationships in the tpos-pdl1t and tpos-um-pdl1t groups differ from those in the tpos-umt group, indicating that these common differential gut microbiota and KEGG pathways may mediate the mechanisms of PD-L1 inhibitor immunotherapy in tumor treatment.

### 3.5. Effects of Different Tumor Intervention Schemes on KEGG Metabolic Pathway of Gut Microbiota in Mice

**Figure 5:**
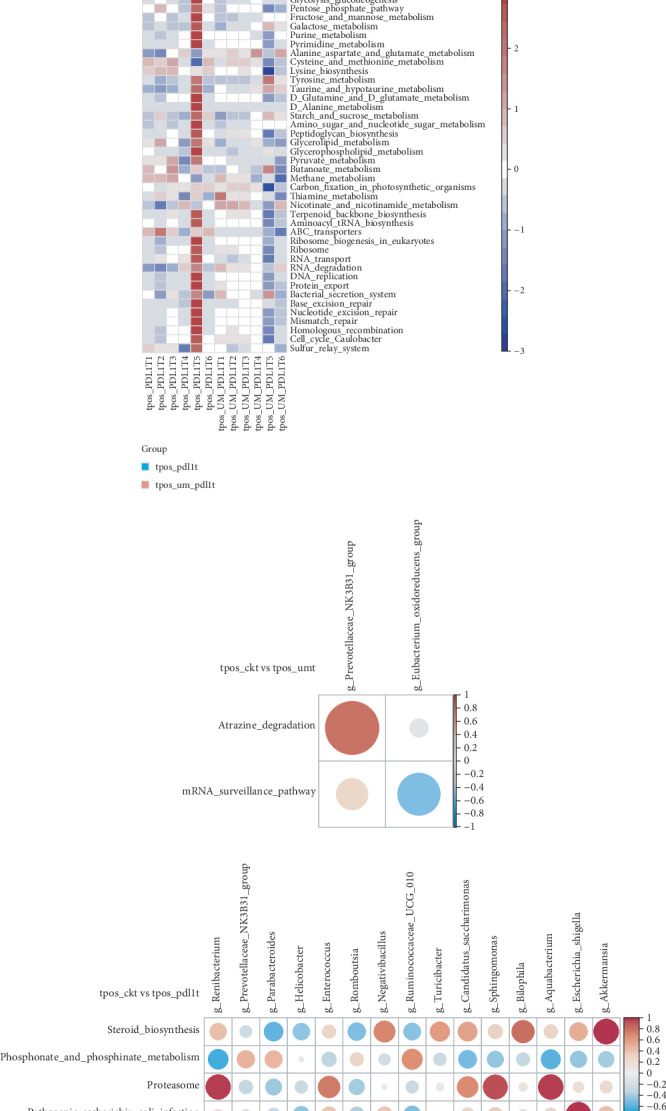
Changes of metabolic pathway of intestinal flora in mice before and after treatment with different tumor intervention schemes. (a–f) KEGG pathway of differentially altered metabolites between different tumor intervention schemes. (g–l) Correlation heatmap of differentially abundant gut microbiota and differentially altered metabolites (*p* < 0.05) from different tumor intervention schemes. Blue represents a positive correlation, red represents a negative correlation, and a darker color corresponds to a stronger correlation. ⁣^∗^*p* < 0.05, ⁣^∗∗^*p* < 0.01, and ⁣^∗∗∗^*p* < 0.001.

To identify gut metabolite pathways potentially modulated by microbiome alterations, we conducted KEGG pathway analysis to assess the relative abundance of microbiota-associated metabolic pathways. As shown in Figures 5a, 5b, 5c, 5d, 5e, and 5f, steroid biosynthesis, steroid hormone biosynthesis, proteasome activity, pathogenic *Escherichia coli* infection, tryptophan metabolism, beta-alanine metabolism, caprolactam degradation, mRNA surveillance pathway, NOD-like receptor signaling pathway, bacterial invasion of epithelial cells, folate biosynthesis, protein digestion and absorption, and carotenoid biosynthesis were all elevated in the tpos-pdl1t and tpos-um-pdl1t groups. Conversely, phosphonate and phosphinate metabolism, oxidative phosphorylation, penicillin and cephalosporin biosynthesis, and *Staphylococcus aureus* infection were more pronounced in the tpos-ckt and tpos-umt groups. To further delve deeper into the possible links between gut microbiome alterations and metabolic byproducts, we created a correlation matrix employing Spearman's rank correlation analysis (Figures 5g, 5h, 5i, 5j, 5k, and 5l). The dominant metabolic pathways in the tpos-pdl1t and tpos-um-pdl1t groups were significantly positively correlated with *g_Akkermansia*, *g_Bacteroides*, *g_Renibacterium*, *g_Aquabacterium*, *g_Escherichia_Shigella*, *g_Sphingomonas*, *g_Candidatus_Saccharimonas*, *g_Enterococcus*, *g_Bilophila*, and *g_Negativibacillus*. In contrast, the dominant metabolic pathways in the tpos-ckt and tpos-umt groups were significantly positively correlated with *g_Lactobacillus*, *g_Muribaculum*, *g_Parabacteroides*, and *g_Ruminococcaceae_UCG_010*.

**Figure 6:**
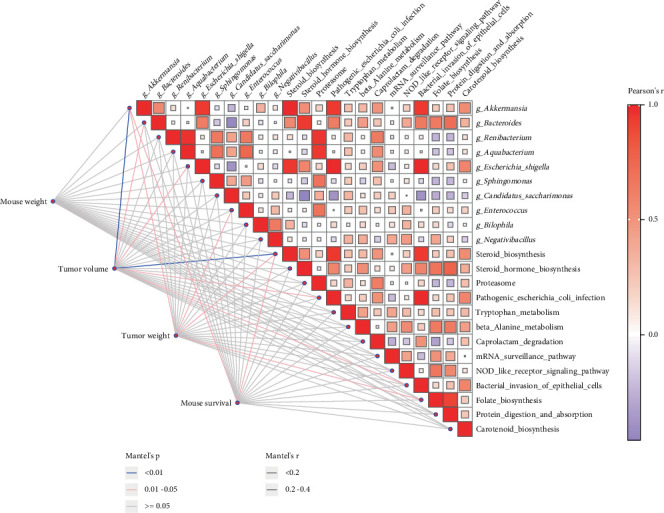
Mantel test. Pairwise comparisons of bacterium and KEGG pathways are shown, with a color gradient denoting Pearson's correlation coefficients. Mouse weight, tumor volume, tumor weight, and mouse survival was related to each bacterium and KEGG pathways factor by partial (geographic distance–corrected) Mantel tests. Edge width corresponds to Mantel's *r* statistic for the corresponding distance correlations, and edge color denotes the statistical significance.

Finally, we conducted matrix correlation analysis using the Mantel test to examine the relationships between body weight, tumor weight, tumor volume, and survival time with significantly different gut microbiota and metabolic pathways across the four groups. This analysis identified four key genera (*g_Akkermansia*, *g_Bacteroides*, *g_Escherichia_Shigella*, and *g_Enterococcus*) and key KEGG pathways (steroid biosynthesis, pathogenic *Escherichia coli* infection, bacterial invasion of epithelial cells, and folate biosynthesis) that may be associated with the efficacy of the tumor intervention schemes (Figure 6). These differential gut bacteria may promote adaptive immunity by regulating the homeostasis of the intestinal environment, improving the tumor microenvironment, and enhancing the antitumor effects of anti-PD-1/PD-L1 therapies. Taken together, these findings suggest that these distinct microbiota and metabolic pathways may be associated with the efficacy of different intervention schemes on MC38 tumors in mice.

## 4. Discussion

This study presents novel insights into the combinatorial effects of USMC and anti-PD-1/PD-L1 immunotherapy on gut microbiome dynamics in MC38 tumor-bearing mice. We have initially found that both the timing of tumor transplantation and the treatment schemes involving PD-L1 inhibitor alone and USMC combined with PD-L1 inhibitor had significant effects on the gut microbiota in mice. Additionally, we aimed to identify microbiota biomarkers associated with the varying responses to different cancer treatment strategies.

We preliminarily found that the gut microbiota of tumor-bearing mice exhibited significant changes. In contrast to mice without tumors, those with tumors exhibited a notably diminished *α* diversity in their gut microbiome, and the dynamics within the microbial ecosystem became increasingly intricate, characterized by heightened interspecies interactions. Notably, negative correlations among these species increased significantly, and as the tumor progressed, the complexity of the microbial community relationships also intensified. Interventions with anti-PD-1/PD-L1 or USMC combined with anti-PD-1/PD-L1 partially reversed the deterioration of the gut microbiota in tumor-bearing mice. In the pos-ckt and tpos-ckt groups, the Bacteroidetes phylum was enriched, representing one of the major phyla within the colonic microbiota. Studies have indicated that members of this phylum can limit inflammation by stimulating the differentiation of T-regulatory cells [36, 37]. At the genus level, *g_Anaeroplasma*, *g_Staphylococcus*, *g_Lactococcus*, *g_Candidatus_Arthromitus*, and *g_Dubosiella* were significantly enriched in the pos-ckt group. These genera are primarily involved in metabolic pathways related to lipopolysaccharide biosynthesis, primary bile acid biosynthesis, and secondary bile acid biosynthesis. Previous research has shown that in patients with non–small cell lung cancer (NSCLC) undergoing concurrent chemoradiotherapy, lipopolysaccharide biosynthesis was overrepresented in those with short progression-free survival (short-PFS) [38]. Bacterial lipopolysaccharides increase intestinal tight junction permeability and play a crucial role in both intestinal and systemic inflammatory responses [39]. Additionally, the lipopolysaccharide biosynthesis pathway is significantly enriched in the gut microbiota of patients with noncachectic cancer [40]. Moreover, studies have demonstrated that bile acids serve a dual role as signaling molecules for gut microbes, influencing the concentration of liver NKT cells through the upregulation of CXCL16, thereby enhancing antitumor responses against both primary and secondary liver malignancies [41]. Suppressing the synthesis of primary bile acids could play a substantial role in the development, advancement, and unfavorable outcomes of hepatocellular carcinoma (HCC), thus presenting a promising avenue for therapeutic intervention in HCC [42]. The genetic deletion of the Farnesoid X receptor (FXR), a natural receptor for bile acids, results in increased bile acid levels and promotes the development of liver tumors in mice [43, 44]. Bile acids can directly damage cell membranes, activate the PKC-MAPK-NF-*κ*B signaling cascade, and increase levels of cytokines such as TNF-*α*, IL-1*β*, and IL-6. These cytokines promote the survival of DNA-damaged cells by activating the JAK-STAT3 and PI3K-MDM2 pathways, contributing to HCC development [42]. Furthermore, bile acid–induced membrane damage can increase intracellular reactive oxygen species (ROS) levels through the activation of cytoplasmic phospholipase A2 (PLA2), which directly activates NF-*κ*B and can also induce cellular DNA damage, potentially leading to HCC [45]. This highlights the pivotal role of bile acids in modulating the immune context of the tumor microenvironment. In our study, we found that metabolic pathways concerning the synthesis of lipopolysaccharide, as well as primary and secondary bile acids, along with the bacteria linked to these processes, were markedly increased in mice with tumors. However, after treatment with PD-L1 inhibitor or USMC combined with PD-L1 inhibitor, these metabolic pathways and their associated gut microbiota were significantly reduced, suggesting that the intestinal flora is associated with the efficacy of PD-L1 inhibitor and USMC combined with PD-L1 inhibitor treatment on tumors.

Previous studies have shown that *Bifidobacterium* spp. was significantly more prevalent in patients with metastatic melanoma who responded to anti-PD-1 blockade and could elicit antitumor immunity [19, 22]. In contrast, another study indicated that *Ruminococcaceae* and *Faecaliaceae* were significantly more prevalent in patients who responded to metastatic melanoma treatment [21]. Additionally, *Akkermansia muciniphila* has been associated with favorable outcomes following PD-1 blockade in epithelial tumors [10, 46]. Jin et al. enrolled 37 Chinese patients with advanced NSCLC treated with nivolumab, and sequencing revealed an enrichment of *Alistipes putredinis*, *Bifidobacterium longum*, and *Prevotella copri* in responding patients, while *Ruminococcus unclassified* was enriched in nonresponding patients [47]. The findings imply that the immune-modulating function of the gut microbiome is subject to variation based on ethnicity, geographical location, and the specific type of cancer.

In this study, we analyzed the efficacy and gut microbiota associated with different tumor intervention strategies. We preliminarily found that treatment with anti-PD-1/PD-L1 alone, or in combination with USMC before and after intervention, resulted in significant differences in both efficacy and gut microbiota compared to the ck and untreated (um) groups. The tumor progression rates did not exhibit a substantial difference between the um and ck groups; however, tumor volume and weight were significantly reduced, and the survival time of the mice was notably improved following treatment with anti-PD-1/PD-L1 alone or in combination with USMC. After treatment with either anti-PD-1/PD-L1 or USMC combined with anti-PD-1/PD-L1, the gut microbiota of the mice showed significant improvement, with notable enrichment of several beneficial symbiotic bacteria, including *Akkermansia*, *Lactobacillus*, *Escherichia-Shigella*, *Enterococcus*, and *Bacteroides*. Previous studies have indicated that *LGG* can induce cGAS/STING-dependent Type I interferon, enhancing the efficacy of immune checkpoint inhibitors [48]. Zhou et al. demonstrated that ginseng polysaccharides enhance host immune function by promoting the proliferation of two predominant metabolic microbes, *Lactobacillus* spp. and *Bacteroides* spp., thereby alleviating symptoms of over fatigue and acute cold stress [49]. *Akkermansia* is a symbiotic genus within the *Verrucomicrobia* phylum that degrades mucin [50] and is a prominent member of the human gut microbiota [51]. Its establishment in the gut has been documented to offer a safeguard against obesity stemming from dietary factors [52, 53], promote mucosal wound healing [54], induce adaptive immune responses during intestinal stabilization [32], and enhance antitumor responses during anti-PD-1 immunotherapy [10], which is consistent with our research results. *Escherichia-Shigella* and *Akkermansia* exhibit mutual symbiosis, with increases in *Escherichia-Shigella* further promoting the enrichment of *Akkermansia*. According to previous research, *Enterococcus* can activate immune signaling pathways, regulate infections [27–29], influence autoimmunity [30], enhance the efficacy of checkpoint inhibitor immunotherapy [55], and impact graft-versus-host disease [31]. KEGG pathway analysis revealed that these beneficial symbiotic bacteria were strongly associated with various metabolic pathways, including steroid biosynthesis, pathogenic *Escherichia coli* infection, bacterial invasion of epithelial cells, and folate biosynthesis. Notably, studies have suggested that folate deficiency may increase the risk of colorectal cancer, particularly in the presence of precancerous lesions [56]. In conclusion, different tumor intervention strategies can significantly alter the gut microbiota of tumor-bearing mice, and conversely, variations in gut microbiota are associated with the efficacy of these tumor intervention strategies.

The strength of this study lies in its status as the first to explore changes in gut microbiota before and after USMC combined with PD-L1 inhibitor immunotherapy for tumor treatment. However, this study also has certain limitations. First, this study was observational in nature, and without GF mice or fecal microbiota transplantation (FMT), the causal role of microbiota in treatment efficacy cannot be established. Second, while our study identified significant microbiota changes, the small sample size (*n* = 6 per group) limits the generalizability of these findings and may have underestimated the true effect size. These limitations are anticipated to be addressed in future research.

## 5. Conclusions

In summary, our results preliminarily indicated that the occurrence and progression of tumors can significantly impact the gut microbiota in mice. Through a comparative analysis of the gut microbiota in mice subjected to different tumor intervention strategies, we identified four distinct microbial populations that may play a crucial role in the effectiveness of anti-PD-1/PD-L1 treatment for tumors. Based on previous studies, we hypothesized that the underlying mechanism may involve the induction of adaptive immunity, which enhances the antitumor effects of anti-PD-1/PD-L1 by regulating intestinal environmental homeostasis. A comprehensive analysis of symbiotic bacteria associated with anticancer and immunotherapy-promoting responses revealed a clear functional connection between microbial ecosystems and anticancer immune surveillance and therapy. Given the current data, regulating the gut microbiota may represent a potential strategy to improve the response rate to tumor immunotherapy.

## Data Availability

The 16S rRNA gene sequence data can be found here: https://bigd.big.ac.cn/gsa/browse/CRA011626.

## Supporting Information

Supporting Information 1 Figure S1: Differences in gut microbiota between mice with different tumor interventions. (a–f) The ANOSIM test outcomes indicate the degree of dissimilarity between group comparisons; intragroup variations are also considered. Greater disparities are reflected by larger distances, while the thickness of the lines corresponds to the sample size. (g–l) The mean percentages of each community contributed by the indicated genus. (m–r) LDA scores indicated varying levels of bacterial taxa abundance among distinct tumor treatment strategies (LDA score > 2.5). ⁣^∗^*p* <0.05, ⁣^∗∗^*p* <0.01, and ⁣^∗∗∗^*p* <0.001.

Supporting Information 2 Table S1: Comparison of mouse weight, tumor volume, tumor weight, and mouse survival between different treatment groups.

## References

[1] Hodi F. S., O'Day S. J., Mcdermott D. F. (2010). Improved Survival With Ipilimumab in Patients With Metastatic Melanoma. *New England Journal of Medicine*.

[2] Hamid O., Robert C., Daud A. (2013). Safety and Tumor Responses With Lambrolizumab (Anti-PD-1) in Melanoma. *New England Journal of Medicine*.

[3] Robert C., Thomas L., Bondarenko I. (2011). Ipilimumab Plus Dacarbazine for Previously Untreated Metastatic Melanoma. *New England Journal of Medicine*.

[4] Restifo N. P., Dudley M. E., Rosenberg S. A. (2012). Adoptive Immunotherapy for Cancer: Harnessing the T Cell Response. *Nature Reviews Immunology*.

[5] Borghaei H., Paz-Ares L., Horn L. (2015). Nivolumab Versus Docetaxel in Advanced Nonsquamous Non-Small-Cell Lung Cancer. *New England Journal of Medicine*.

[6] Topalian S. L., Hodi F. S., Brahmer J. R. (2012). Safety, Activity, and Immune Correlates of Anti-PD-1 Antibody in Cancer. *New England Journal of Medicine*.

[7] Ribas A., Hamid O., Daud A. (2016). Association of Pembrolizumab With Tumor Response and Survival Among Patients With Advanced Melanoma. *JAMA*.

[8] Ribas A., Wolchok J. D. (2018). Cancer Immunotherapy Using Checkpoint Blockade. *Science*.

[9] Pardoll D. (2015). Cancer and the Immune System: Basic Concepts and Targets for Intervention. *Seminars in Oncology*.

[10] Routy B., Le Chatelier E., Derosa L. (2018). Gut Microbiome Influences Efficacy of PD-1–Based Immunotherapy Against Epithelial Tumors. *Science*.

[11] Carney C. P., Pandey N., Kapur A., Woodworth G. F., Winkles J. A., Kim A. J. (2021). Harnessing Nanomedicine for Enhanced Immunotherapy for Breast Cancer Brain Metastases. *Drug Delivery and Translational Research*.

[12] Karthikesh M. S., Yang X. (2021). The Effect of Ultrasound Cavitation on Endothelial Cells. *Experimental Biology and Medicine*.

[13] Yin Y. X. L. H., Jiang X., Sun L. (2021). Continuous Inertial Cavitation Evokes Massive ROS for Reinforcing Sonodynamic Therapy and Immunogenic Cell Death Against Breast Carcinoma. *Nano Today*.

[14] Sun L., Cao Y., Lu Z. (2022). A Hypoxia-Irrelevant Fe-Doped Multivalent Manganese Oxide Sonosensitizer via a Vacancy Engineering Strategy for Enhanced Sonodynamic Therapy. *Nano Today*.

[15] Liu Z., Zhang J., Tian Y. (2018). Targeted Delivery of Reduced Graphene Oxide Nanosheets Using Multifunctional Ultrasound Nanobubbles for Visualization and Enhanced Photothermal Therapy. *International Journal of Nanomedicine*.

[16] Qiao K., Luo C., Huang R. (2023). Ultrasound Triggered Tumor Metabolism Suppressor Induces Tumor Starvation for Enhanced Sonodynamic Immunotherapy of Breast Cancer. *International Journal of Nanomedicine*.

[17] Li N., Tang J., Yang J. (2021). Tumor Perfusion Enhancement by Ultrasound Stimulated Microbubbles Potentiates PD-L1 Blockade of MC38 Colon Cancer in Mice. *Cancer Letters*.

[18] Vétizou M., Pitt J. M., Daillère R. (2015). Anticancer Immunotherapy by CTLA-4 Blockade Relies on the Gut Microbiota. *Science*.

[19] Sivan A., Corrales L., Hubert N. (2015). Commensal Bifidobacterium Promotes Antitumor Immunity and Facilitates Anti-PD-L1 Efficacy. *Science*.

[20] Frankel A. E., Coughlin L. A., Kim J. (2017). Metagenomic Shotgun Sequencing and Unbiased Metabolomic Profiling Identify Specific Human Gut Microbiota and Metabolites Associated With Immune Checkpoint Therapy Efficacy in Melanoma Patients. *Neoplasia*.

[21] Gopalakrishnan V., Spencer C. N., Nezi L. (2018). Gut Microbiome Modulates Response to Anti-PD-1 Immunotherapy in Melanoma Patients. *Science*.

[22] Matson V., Fessler J., Bao R. (2018). The Commensal Microbiome Is Associated With Anti-PD-1 Efficacy in Metastatic Melanoma Patients. *Science*.

[23] Peters B. A., Wilson M., Moran U. (2019). Relating the Gut Metagenome and Metatranscriptome to Immunotherapy Responses in Melanoma Patients. *Genome Medicine*.

[24] Wang Y., Liu L., Moore D. J. (2017). An LGG-Derived Protein Promotes IgA Production Through Upregulation of APRIL Expression in Intestinal Epithelial Cells. *Mucosal Immunology*.

[25] Seow S. W., Cai S., Rahmat J. N. (2010). Lactobacillus rhamnosus GG Induces Tumor Regression in Mice Bearing Orthotopic Bladder Tumors. *Cancer Science*.

[26] Li J., Sung C. Y., Lee N. (2016). Probiotics Modulated Gut Microbiota Suppresses Hepatocellular Carcinoma Growth in Mice. *Proceedings of the National Academy of Sciences of the United States of America*.

[27] Rangan K. J., Pedicord V. A., Wang Y. C. (2016). A Secreted Bacterial Peptidoglycan Hydrolase Enhances Tolerance to Enteric Pathogens. *Science*.

[28] Pedicord V. A., Lockhart A., Rangan K. J. (2016). Exploiting a Host-Commensal Interaction to Promote Intestinal Barrier Function and Enteric Pathogen Tolerance. *Science Immunology*.

[29] Kim B., Wang Y. C., Hespen C. W. (2019). Enterococcus faecium Secreted Antigen a Generates Muropeptides to Enhance Host Immunity and Limit Bacterial Pathogenesis. *eLife*.

[30] Manfredo V. S., Hiltensperger M., Kumar V. (2018). Translocation of a Gut Pathobiont Drives Autoimmunity in Mice and Humans. *Science*.

[31] Stein-Thoeringer C. K., Nichols K. B., Lazrak A. (2019). Lactose Drives Enterococcus Expansion to Promote Graft-Versus-Host Disease. *Science*.

[32] Ansaldo E., Slayden L. C., Ching K. L. (2019). Akkermansia muciniphila Induces Intestinal Adaptive Immune Responses During Homeostasis. *Science*.

[33] Liu P., Wang X., Zhou S., Hua X., Liu Z., Gao Y. (2011). Effects of a Novel Ultrasound Contrast Agent With Long Persistence on Right Ventricular Pressure: Comparison With SonoVue. *Ultrasonics*.

[34] Li H., Guo W., Li S. (2023). Alteration of the Gut Microbiota Profile in Children With Autism Spectrum Disorder in China. *Frontiers in Microbiology*.

[35] Yilmaz B., Juillerat P., Øyås O. (2019). Microbial Network Disturbances in Relapsing Refractory Crohn's Disease. *Nature Medicine*.

[36] Round J. L., Mazmanian S. K. (2010). Inducible Foxp3+ Regulatory T-cell Development by a Commensal Bacterium of the Intestinal Microbiota. *Proceedings of the National Academy of Sciences of the United States of America*.

[37] Faith J. J., Ahern P. P., Ridaura V. K., Cheng J., Gordon J. I. (2014). Identifying Gut Microbe-Host Phenotype Relationships Using Combinatorial Communities in Gnotobiotic Mice. *Science Translational Medicine*.

[38] Qiu B., Xi Y., Liu F. (2023). Gut Microbiome Is Associated With the Response to Chemoradiotherapy in Patients With Non-Small Cell Lung Cancer. *International Journal of Radiation Oncology • Biology • Physics*.

[39] Guo S., Nighot M., Al-Sadi R., Alhmoud T., Nighot P., Ma T. Y. (2015). Lipopolysaccharide Regulation of Intestinal Tight Junction Permeability Is Mediated by TLR4 Signal Transduction Pathway Activation of FAK and MyD88. *Journal of Immunology*.

[40] Ni Y., Lohinai Z., Heshiki Y. (2021). Distinct Composition and Metabolic Functions of Human Gut Microbiota Are Associated With Cachexia in Lung Cancer Patients. *ISME Journal*.

[41] Tomiyama Y., Takenaka K., Kodama T. (2013). Risk Factors for Survival and the Development of Hepatocellular Carcinoma in Patients With Primary Biliary Cirrhosis. *Internal Medicine*.

[42] Zhang T., Nie Y., Gu J. (2021). Identification of Mitochondrial-Related Prognostic Biomarkers Associated With Primary Bile Acid Biosynthesis and Tumor Microenvironment of Hepatocellular Carcinoma. *Frontiers in Oncology*.

[43] Yang F., Huang X., Yi T., Yen Y., Moore D. D., Huang W. (2007). Spontaneous Development of Liver Tumors in the Absence of the Bile Acid Receptor Farnesoid X Receptor. *Cancer Research*.

[44] Kim I., Morimura K., Shah Y., Yang Q., Ward J. M., Gonzalez F. J. (2007). Spontaneous Hepatocarcinogenesis in Farnesoid X Receptor-Null Mice. *Carcinogenesis*.

[45] Sánchez B. (2018). Bile Acid-Microbiota Crosstalk in Gastrointestinal Inflammation and Carcinogenesis: A Role for Bifidobacteria and Lactobacilli?. *Nature Reviews Gastroenterology & Hepatology*.

[46] Huang J., Liu D., Wang Y. (2022). Ginseng Polysaccharides Alter the Gut Microbiota and Kynurenine/Tryptophan Ratio, Potentiating the Antitumour Effect of Antiprogrammed Cell Death 1/Programmed Cell Death Ligand 1 (Anti-PD-1/PD-L1) Immunotherapy. *Gut*.

[47] Jin Y., Dong H., Xia L. (2019). The Diversity of Gut Microbiome is Associated With Favorable Responses to Anti-Programmed Death 1 Immunotherapy in Chinese Patients With NSCLC. *Journal of Thoracic Oncology*.

[48] Si W., Liang H., Bugno J. (2022). *Lactobacillus rhamnosus* GG Induces cGAS/STING- Dependent Type I Interferon and Improves Response to Immune Checkpoint Blockade. *Gut*.

[49] Zhou S. S., Xu J., Zhu H. (2016). Gut Microbiota-Involved Mechanisms in Enhancing Systemic Exposure of Ginsenosides by Coexisting Polysaccharides in Ginseng Decoction. *Scientific Reports*.

[50] Derrien M., Vaughan E. E., Plugge C. M., de Vos W. M. (2004). *Akkermansia muciniphila* gen. nov., sp. nov., a Human Intestinal Mucin-Degrading Bacterium. *International Journal of Systematic and Evolutionary Microbiology*.

[51] Derrien M., Collado M. C., Ben-Amor K., Salminen S., de Vos W. M. (2008). The Mucin Degrader *Akkermansia muciniphila* is an Abundant Resident of the Human Intestinal Tract. *Applied and Environmental Microbiology*.

[52] Everard A., Belzer C., Geurts L. (2013). Cross-Talk Between Akkermansia muciniphila and Intestinal Epithelium Controls Diet-Induced Obesity. *Proceedings of the National Academy of Sciences of the United States of America*.

[53] Greer R. L., Dong X., Moraes A. C. (2016). *Akkermansia muciniphila* Mediates Negative Effects of IFN*γ* on Glucose Metabolism. *Nature Communications*.

[54] Alam A., Leoni G., Quiros M. (2016). The Microenvironment of Injured Murine Gut Elicits a Local Pro-Restitutive Microbiota. *Nature Microbiology*.

[55] Griffin M. E., Espinosa J., Becker J. L. (2021). *Enterococcus* peptidoglycan Remodeling Promotes Checkpoint Inhibitor Cancer Immunotherapy. *Science*.

[56] Kok D. E., Steegenga W. T., Smid E. J., Zoetendal E. G., Ulrich C. M., Kampman E. (2020). Bacterial Folate Biosynthesis and Colorectal Cancer Risk: More Than Just a Gut Feeling. *Critical Reviews in Food Science and Nutrition*.

