# Supplementary figures and images for: Ultrasound-Stimulated Microbubble Cavitation Combined With Anti-PD-L1 Blockade Inhibits the Progression of MC38 Tumors and Alters the Composition of Gut Microbiota in Mice
Source: Int J Microbiol. 2025 Oct 17;2025:5514372. doi: 10.1155/ijm/5514372 (PMC12552084; doi:10.1155/ijm/5514372)

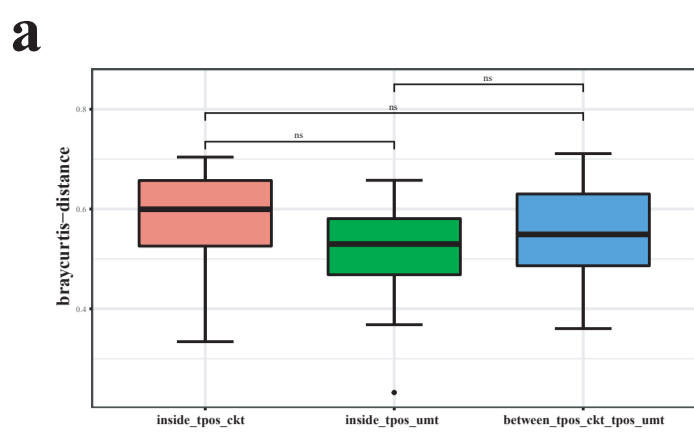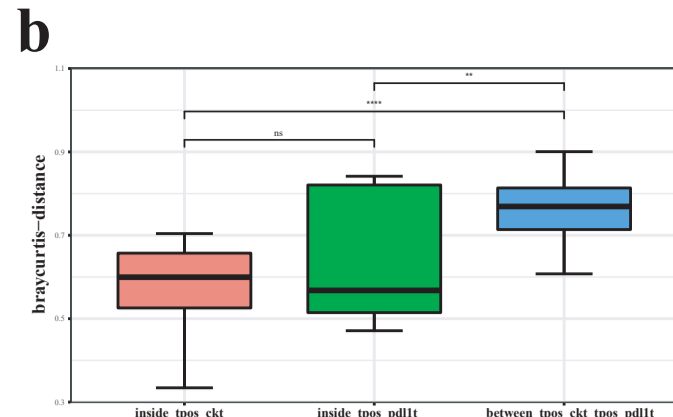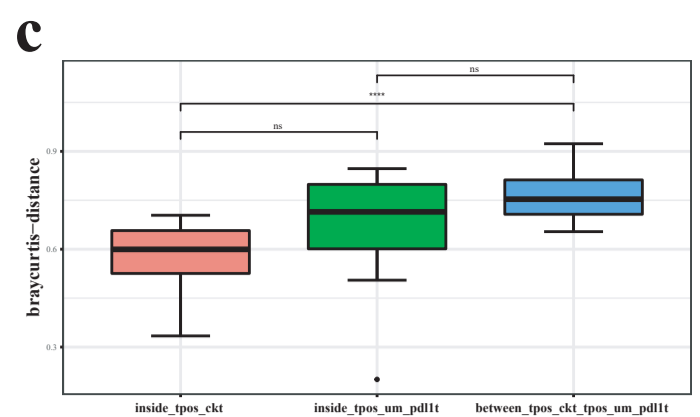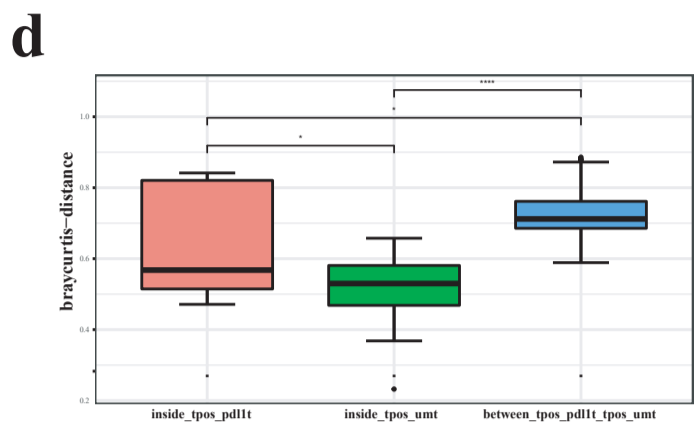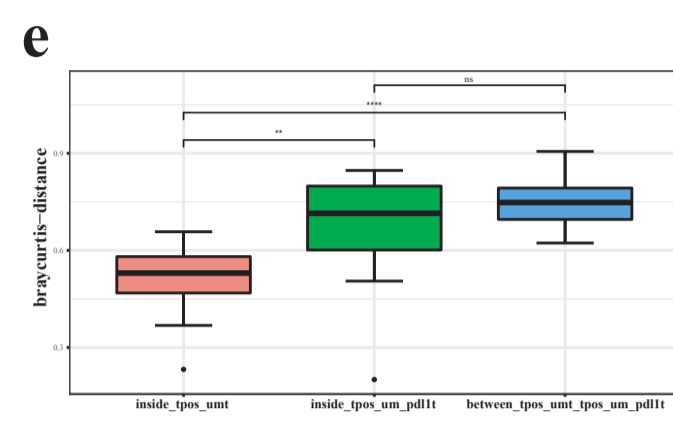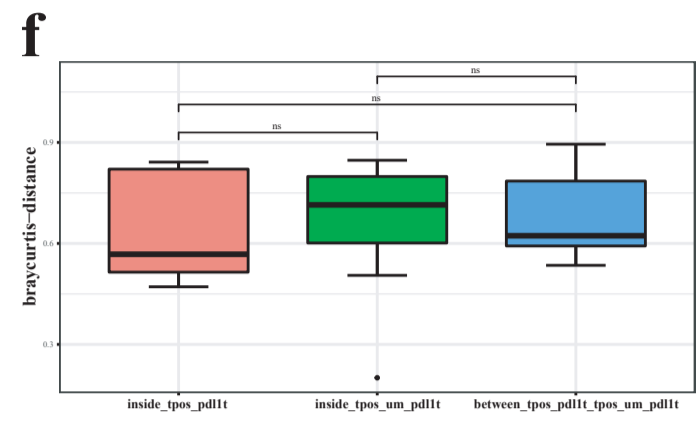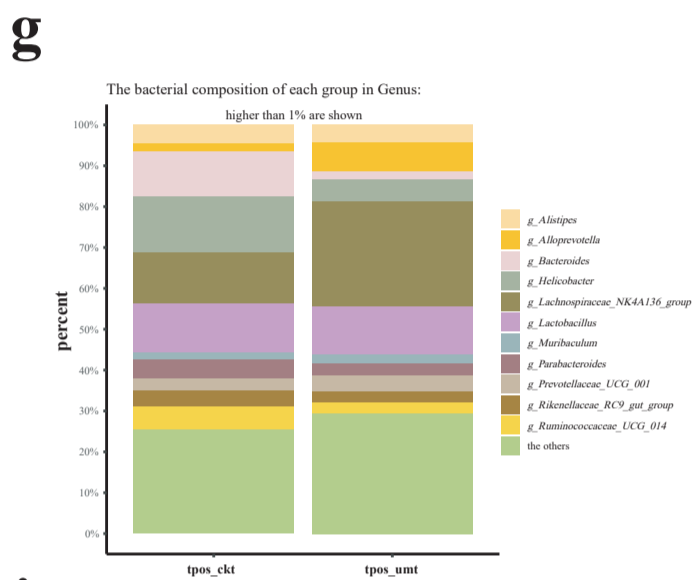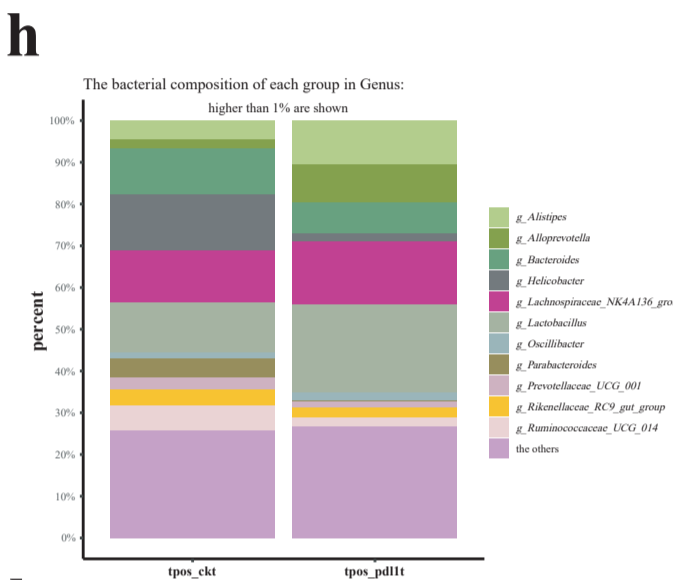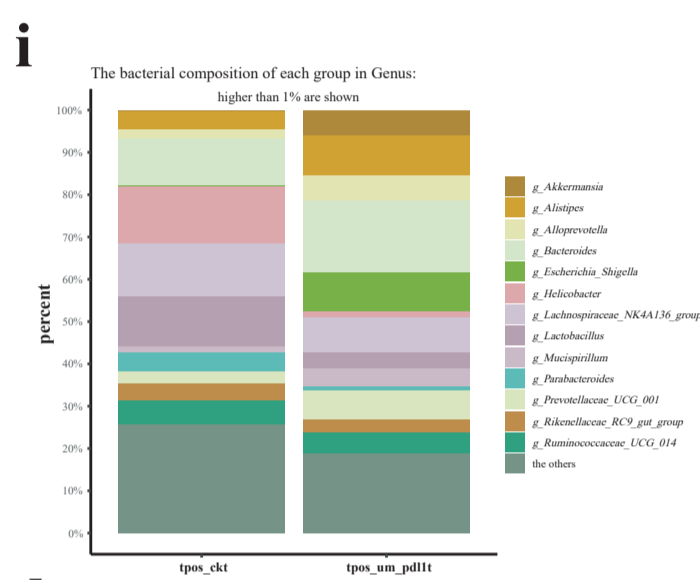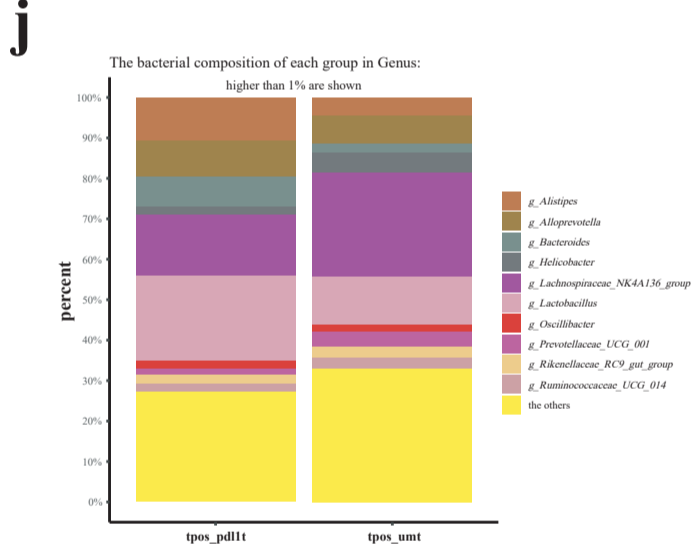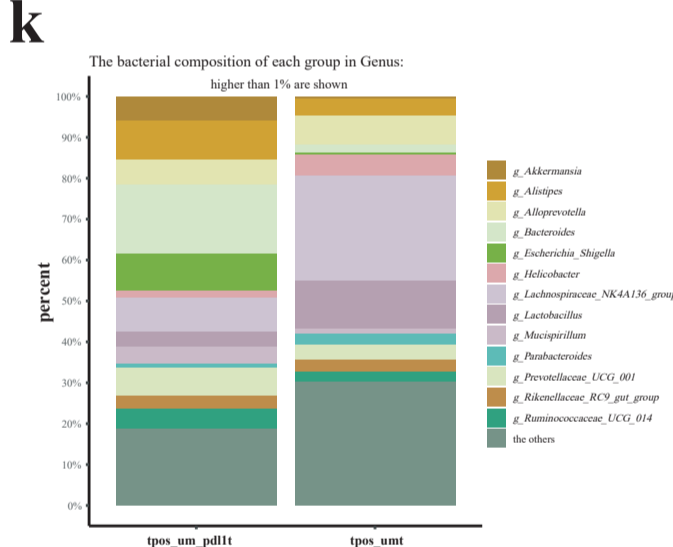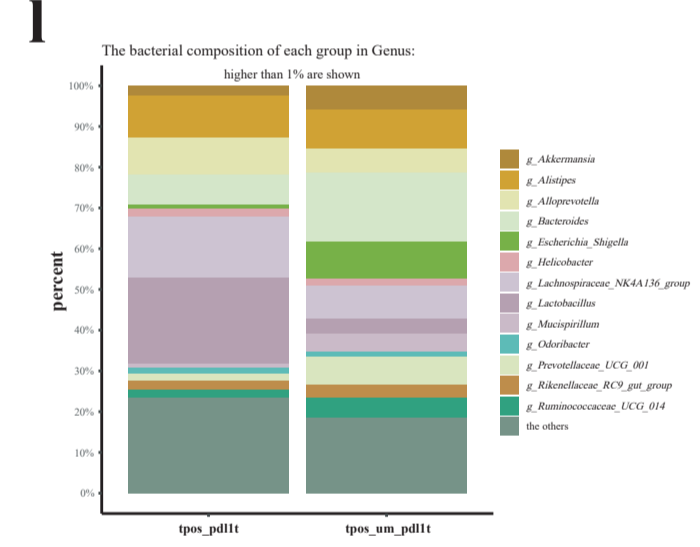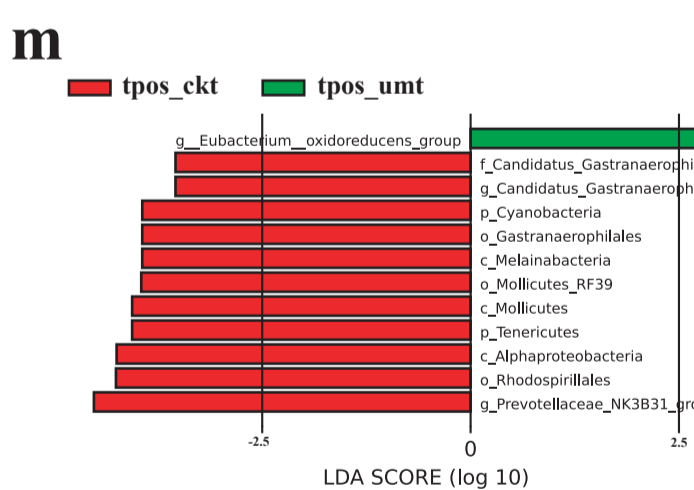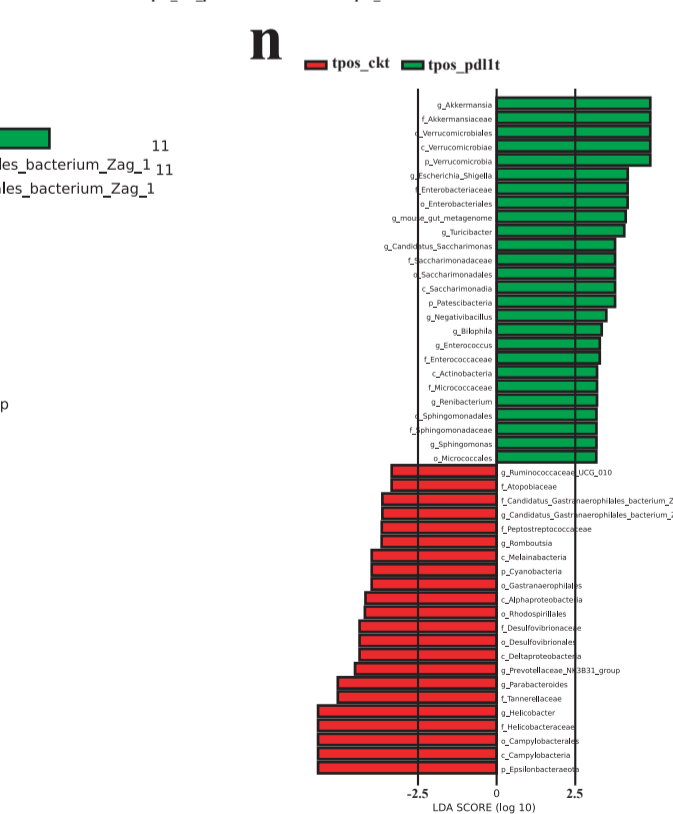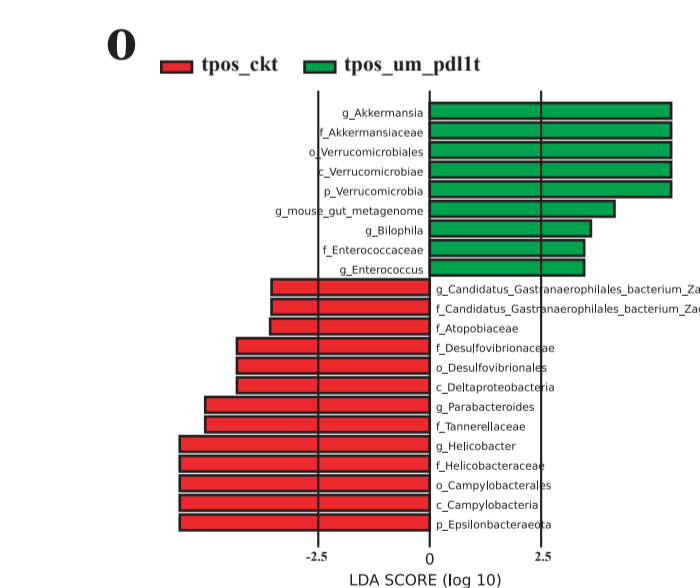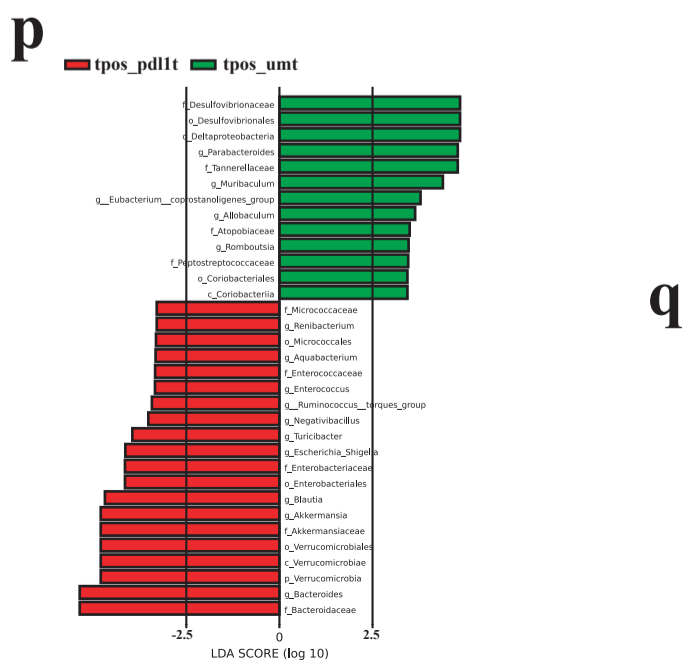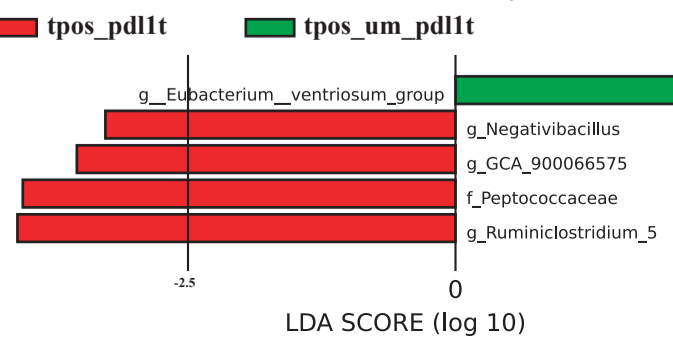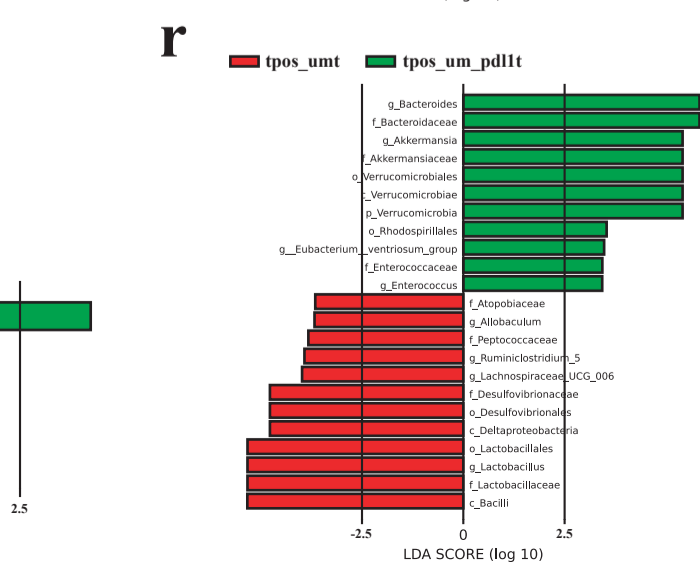

Supplement: Supporting Information 1 — Figure S1: Differences in gut microbiota between mice with different tumor interventions. (a–f) The ANOSIM test outcomes indicate the degree of dissimilarity between group comparisons; intragroup variations are also considered. Greater disparities are reflected by larger distances, while the thickness of the lines corresponds to the sample size. (g–l) The mean percentages of each community contributed by the indicated genus. (m–r) LDA scores indicated varying levels of bacterial taxa abundance among distinct tumor treatment strategies (LDA score > 2.5). ⁣∗p <0.05, ⁣∗∗p <0.01, and ⁣∗∗∗p <0.001. [file 5514372.f1.pdf]
