# Supplementary material for: Ultrasound-Stimulated Microbubble Cavitation Combined With Anti-PD-L1 Blockade Inhibits the Progression of MC38 Tumors and Alters the Composition of Gut Microbiota in Mice
Source: Int J Microbiol. 2025 Oct 17;2025:5514372. doi: 10.1155/ijm/5514372 (PMC12552084; doi:10.1155/ijm/5514372)
Supplement: Supporting Information 2 — Table S1: Comparison of mouse weight, tumor volume, tumor weight, and mouse survival between different treatment groups. [file 5514372.f2.docx]

**Table 1 Comparison of mouse weight, tumor volume, tumor weight and mouse survival between different treatment Groups**

| Group | Sample ID | Mouse Weight/g | Tumor Volume/mm^3^ | Tumor Weight/g | Mouse Survival/day |
| --- | --- | --- | --- | --- | --- |
| tpos_ckt | tpos_ckt1 | 19.91 | 918.72 | 0.69 | 34 |
|  | tpos_ckt2 | 19.28 | 1239.34 | 0.71 | 34 |
|  | tpos_ckt3 | 20.73 | 1029.29 | 0.79 | 29 |
|  | tpos_ckt4 | 20.04 | 1236.75 | 0.75 | 30 |
|  | tpos_ckt5 | 19.87 | 1219.92 | 0.68 | 38 |
|  | tpos_ckt6 | 20.32 | 1267.16 | 0.79 | 44 |
| tpos_umt | tpos_umt1 | 18.64 | 1056.59 | 0.67 | 44 |
|  | tpos_umt2 | 19.66 | 1205.63 | 0.66 | 46 |
|  | tpos_umt3 | 19.08 | 950.42 | 0.7 | 30 |
|  | tpos_umt4 | 18.79 | 942.6 | 0.77 | 36 |
|  | tpos_umt5 | 20.21 | 1095.94 | 0.67 | 42 |
|  | tpos_umt6 | 18.83 | 884.52 | 0.8 | 39 |
| tpos_pdl1t | tpos_pdl1t1 | 19.88 | 702.39 | 0.45 | 59 |
|  | tpos_pdl1t2 | 23.12 | 601.01 | 0.5 | 62 |
|  | tpos_pdl1t3 | 19.75 | 628.12 | 0.41 | 61 |
|  | tpos_pdl1t4 | 20.36 | 675.67 | 0.39 | 41 |
|  | tpos_pdl1t5 | 20.69 | 592.15 | 0.47 | 53 |
|  | tpos_pdl1t6 | 20.45 | 673.19 | 0.51 | 64 |
| tpos_um_pdl1t | tpos_um_pdl1t1 | 19.47 | 188.78 | 0.32 | 66 |
|  | tpos_um_pdl1t2 | 19.89 | 205.7 | 0.47 | 70 |
|  | tpos_um_pdl1t3 | 21.62 | 199.19 | 0.29 | 46 |
|  | tpos_um_pdl1t4 | 18.12 | 199.42 | 0.36 | 70 |
|  | tpos_um_pdl1t5 | 18.98 | 204.99 | 0.37 | 70 |
|  | tpos_um_pdl1t6 | 19.34 | 204.14 | 0.33 | 70 |
